# 

**Published:** 1920-06

**Authors:** 


### ANNOUNCEMENT

---

MEETING OF THE NATIONAL DENTAL ASSOCIATION

The twenty-fourth annual meeting will be held in Boston, August 23 to 27, 1920.

The meeting will be largely attended and a crowded time of the year will mean a demand for hotel accommodations that will not be easily cared for. Dr. William Rice is chairman of hotel reservations and all applications should be sent to him at an early date. The *National Dental Journal* is issuing full particular announcements. By a special arrangement all reservations will be made by the committee. Any one expecting to attend should make complete reservations at once.

